# „Polypill“ in der kardiovaskulären Prävention – erfolgreich durch Vereinfachung? Neue Studienergebnisse zum Nutzen der Polypill-Strategie in der Primär- und Sekundärprävention

**DOI:** 10.1007/s00108-023-01506-0

**Published:** 2023-05-25

**Authors:** Patrick Despang, Martin Schikora, Wolfram Doehner

**Affiliations:** 1 Medizinische Abteilung, APONTIS PHARMA GmbH & Co. KG, Monheim, Deutschland; 2 Kardiologische Abteilung, Brandenburgklinik, Michels Kliniken, Bernau bei Berlin, Deutschland; 3 Berliner Institut für Gesundheitsforschung – Zentrum für regenerative Therapien (BCRT), Charité – Universitätsmedizin Berlin, Augustenburger Platz 1, 13353 Berlin, Deutschland; 4 Medizinische Klinik mit Schwerpunkt Kardiologie (Virchow-Klinikum), Charité – Universitätsmedizin Berlin, Berlin, Deutschland; 5 Deutsches Zentrum für Herz-Kreislauf-Forschung (DZHK), Standort Berlin, Berlin, Deutschland

**Keywords:** Therapieadhärenz, Compliance, Herz-Kreislauf-Erkrankungen, Medikamentöse Kombinationstherapie, Therapieoutcome, „Polypill“, Treatment adherence, Compliance, Cardiovascular diseases, Drug therapy, combination, Treatment outcome, Polypill

## Abstract

**Hintergrund:**

Herz-Kreislauf-Erkrankungen sind nach wie vor die häufigste Todesursache weltweit. Neben einem erhöhten Blutdruck ist ein weiterer modifizierbarer Risikofaktor ein erhöhtes Low-density-Lipoprotein-Cholesterin. Obwohl beides gut medikamentös kontrollierbar ist, bleibt die Kontrolle bisher mangelhaft. Eine wesentliche Ursache ist eine unzureichende Adhärenz zur Medikation. Eine Lösung hierfür ist das Konzept der „Polypill“, also die Kombination mehrerer Wirkstoffe in einer einzelnen Tablette. Hierdurch wird nicht nur die Therapieadhärenz verbessert, sondern auch eine Verringerung kardiovaskulärer Ereignisse und eine Verbesserung der Prognose der Patienten erreicht.

**Ziel der Übersicht:**

Diese Übersichtsarbeit fasst die aktuellen Evidenzen aus randomisierten klinischen Studien in der Primär- und Sekundärprävention zusammen. Ein wesentlicher Fokus liegt auf der aktuell publizierten SECURE-Studie, die die Wirksamkeit der „Polypill“ in der Sekundärprävention untersucht.

**Datenlage:**

Viele Studien zur „Polypill“ beschäftigen sich mit der Kontrolle der Risikofaktoren und der Verbesserung der Therapieadhärenz, ohne jedoch einen prognostischen Vorteil zu adressieren. Neuere Studien wie HOPE‑3, PolyIran und TIPS‑3 konnten in der Primärprävention einen prognostischen Vorteil aufzeigen. In der Sekundärprävention war dies bis jetzt noch nicht geschehen. Diese Lücke wurde nun durch die SECURE-Studie geschlossen. Hier wurde bei Patienten nach Infarkt nicht nur eine signifikante Reduktion schwerwiegender kardiovaskulärer Ereignisse, sondern auch eine Reduktion kardiovaskulärer Todesfälle durch die „Polypill“ nachgewiesen.

**Schlussfolgerung:**

Das Konzept der „Polypill“ hat sich von einer Komfortmaßnahme – einer Erleichterung der Medikamenteneinnahme für die Patienten – weiterentwickelt hin zu einem innovativen Therapiekonzept mit nachgewiesenem prognostischem Vorteil in Form einer Reduktion schwerwiegender Ereignisse und Todesfälle. Es ist an der Zeit, das Konzept der „Polypill“ breit einzusetzen, um die Bürde der Herz-Kreislauf-Erkrankungen weltweit zur verringern.

## Abstract (en)

**Background:**

Cardiovascular disease is still the major cause of death worldwide. Beside the elevated blood pressure, a major modifiable risk factor is the elevated low-density lipoprotein (LDL) cholesterol. Although both risk factors are well manageable, therapeutic control remains poor with low adherence to medication being a major cause of insufficient treatment success. One solution to overcome this issue is the polypill concept, i.e. a combination of different drugs in one tablet. This not only increases adherence but also significantly improves patients’ prognosis by reducing cardiovascular events.

**Objective:**

This review focuses on current evidence published in randomized control trials in primary and secondary prevention. A major focus is on the recently published SECURE trial dealing with the polypill in secondary prevention.

**Current data:**

Most trials dealing with the polypill concept focus on the control of risk factors such as blood pressure and LDL cholesterol while lacking a prognostic benefit in the form of a reduction in cardiovascular events. Recent trials such as the HOPE‑3, PolyIran and TIPS‑3 trials have shown a prognostic improvement for the polypill in primary prevention. In secondary prevention there has been a lack of prognostic benefit for the polypill to date. The recently published SECURE trial closed this gap by showing a significant reduction in major adverse cardiovascular events in post-infarct patients and also showing a reduction in cardiovascular death by 33%.

**Conclusion:**

The concept of the polypill has evolved from a comfort method for patients aimed at facilitating adherence to an innovative therapeutic concept with a proven prognostic advantage compared to current treatment practice by reducing cardiovascular events and mortality. Therefore, it is time to implement the concept of the polypill in primary and secondary prevention to improve patients’ prognosis and reduce the burden of cardiovascular disease worldwide.

Herz-Kreislauf-Erkrankungen sind weltweit für etwa 18 Mio. Todesfälle jährlich verantwortlich, wobei im Jahr 2019 etwa 10,8 Mio. dieser Todesfälle auf einen erhöhten Blutdruck, den primären Risikofaktor für Herz-Kreislauf-Erkrankungen, zurückzuführen waren [1]. Neben dem Bluthochdruck ist der zweite wesentliche modifizierbare Risikofaktor bei Herz-Kreislauf-Erkrankungen das Vorliegen einer Dyslipidämie in Form eines erhöhten Low-density-Lipoprotein(LDL)-Cholesterins. Wenn beide Risikofaktoren zusammen vorliegen, erhöht sich das kardiovaskuläre Risiko nochmals um das 2‑ bis 3‑fache im Vergleich zum Vorliegen eines Risikofaktors allein [27].

Leitlinien empfehlen für die kardiovaskuläre Prophylaxe eine medikamentöse Kombinationstherapie

Die Kontrolle der Risikofaktoren spielt nach wie vor die übergeordnete Rolle bei der Senkung der Inzidenz von Herz-Kreislauf-Erkrankungen. Neben einer Anpassung des Lebensstils empfehlen nationale sowie internationale Leitlinien in der Primär- wie auch Sekundärprophylaxe eine medikamentöse Therapie bestehend u. a. aus Angiotensin-converting-enzyme(ACE)-Hemmern oder Angiotensinrezeptorblockern (ARB), Betablockern, Kalziumkanalblockern (KKB), Diuretika und Thrombozytenaggregationshemmern. Die aktuellen europäischen und internationalen Leitlinien zur Hypertonie empfehlen hier bereits zur Initialtherapie die Gabe einer Kombination aus entweder ACE-Hemmer oder ARB plus KKB oder Diuretikum, im besten Fall in Form einer einzelnen Tablette als Kombinationspräparat. Als weiterer Eskalationsschritt wird die Gabe eines dritten Antihypertensivums, das in der Initialtherapie noch nicht verabreicht worden ist, empfohlen (entweder KKB oder Diuretikum; [2]).

## Gründe für die Verwendung von Kombinationspräparaten

**Figure 1:**
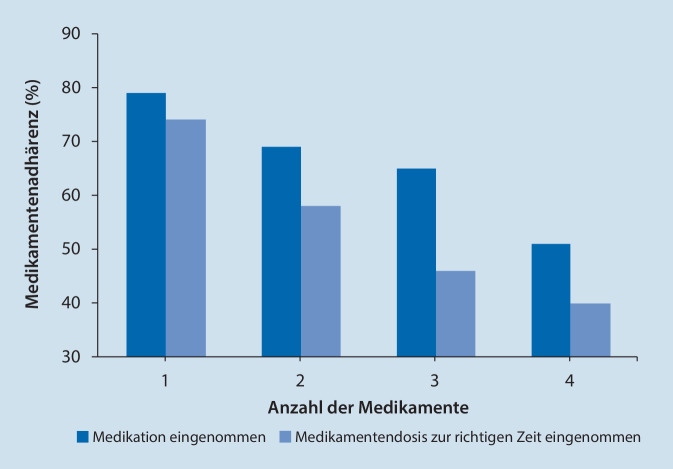

Die Gründe hinter der Empfehlung zur Verordnung von Fixkombinationen sind lange bekannt. Eine steigende Anzahl der einnahmepflichtigen Tabletten ist direkt assoziiert mit dem Rückgang der Einnahmetreue (Adhärenz) hinsichtlich der Medikation [3]. Davon ist sowohl die grundsätzliche Einnahme der Tabletten als auch die zeitlich richtige Dosierung betroffen (Abb. 1).

Auch nach einem einschneidenden Krankheitsereignis wie einem Herzinfarkt lassen etwa 30 % der Patienten bereits 3 Monate nach dem akuten Ereignis häufig eines der verordneten Medikamente weg, die Adhärenzraten liegen zwischen 50 und 79 % [4]. Welche Konsequenzen sich aus einer inkonsequenten Therapie für Patienten mit Herz-Kreislauf-Erkrankungen ergeben, wurde seitdem durch zahlreiche Studien belegt. So führte eine schlechte Therapieadhärenz bei Patienten mit neu diagnostiziertem Bluthochdruck innerhalb der ersten 3 Jahre zu einem erhöhten relativen Risiko (RR) von 7 % für eine Koronarerkrankung, von 13 % für eine zerebrovaskuläre Erkrankung und von 42 % für die Erstmanifestation einer Herzinsuffizienz im Vergleich zu Patienten mit guter Compliance [5]. In einer Registerstudie mit 9998 Patienten lag die Mortalität ein Jahr nach akutem Herzinfarkt bei 4,9 % unter Betablockertherapie und der empfohlenen zusätzlichen Therapie mit Statinen, ACE-Hemmern und Acetylsalicylsäure (ASS). Die Mortalität lag dagegen doppelt so hoch (9,7 %), wenn nur 2 der zusätzlichen Medikamente verordnet wurden und war fast 3‑fach erhöht (13,6 %), wenn 0 oder 1 der zusätzlichen Medikamente verordnet wurden [6]. In der prospektiven FOCUS-Studie konnte gezeigt werden, dass die durchschnittliche Anzahl der einzunehmenden Medikamente nach einem Myokardinfarkt bei 7 ± 3 lag. Hier verbesserte die Gabe einer „Polypill“, die ASS, Ramipril und Atorvastatin enthielt, die Adhärenz sowohl in der Intention-to-treat- als auch in der Per-protocol-Analyse signifikant [7]. Die Bedeutung einer Kombinationstherapie mit einer einzelnen Tablette als Lösungsansatz für mangelhafte Compliance konnten Weisser et al. [8] in einer Metaanalyse zeigen. In allen Vergleichen war die Compliance unter einer Polypill-Strategie höher als unter der identischen losen Kombination einzelner Tabletten.

Eine steigende Zahl einzunehmender Tabletten ist direkt assoziiert mit dem Rückgang der Adhärenz

Ziel dieser Übersichtsarbeit ist es, die aktuelle Evidenzlage bezüglich Fixkombinationen („Single Pills“/„Polypills“) in der Primär- und Sekundärprävention vor allem in Hinblick auf die prognoserelevanten Vorteile zusammenzufassen und die Praxisrelevanz herauszustellen, wobei der Fokus auf randomisierten klinischen Studien liegt.

## Von den Anfängen des Polypill-Konzepts

Die Idee, dass Fixkombinationen mehrerer Wirkstoffe in einer einzigen Tablette einen großen Nutzen bei der Reduktion und Prävention von Herz-Kreislauf-Erkrankungen haben können, wurde erstmals 2003 von Wald u. Law [9] im *British Medical Journal* propagiert. Auf Basis von randomisierten, kontrollierten Studien (RCT) und Metaanalysen schätzten sie, dass das Konzept der Polypill, bestehend aus einem Statin, einem blutdrucksenkenden Mittel in niedriger Dosierung, Folsäure sowie ASS, die Zahl ischämischer Herz-Kreislauf-Erkrankungen um 88 % und die Zahl der Schlaganfälle um 80 % reduzieren könnte.

Ein wichtiger Gedanke hinter der Entwicklung des Polypill-Konzepts war außerdem, in Ländern mit niedrigem Einkommen (Entwicklungs- und Schwellenländer) durch die Gabe von bis zu 3 Wirkstoffen in einer Tablette und die damit einhergehende verbesserte Therapieadhärenz die Primärprävention kostengünstig zu verbessern. Die Verwendung der „Polypill“ wurde generell als eine wirkungsvolle Lösung für Menschen mit schlechtem Zugang zum Gesundheitssystem und begrenzten Ressourcen gesehen, um so eine verbesserte Prävention kardiovaskulärer Ereignisse in der Situation einer unzureichenden Versorgung zu gewährleisten [10]. Grundsätzlich ist das Konzept der Kombination mehrerer Wirkstoffe in einer Tablette oder Kapsel mit verschiedenen Begriffen beschrieben worden, neben „Polypill“ auch mit „Single Pill“, Fixkombination, Fixed-Dose-Präparat oder Kombinationspräparat. Im Weiteren wird in diesem Beitrag einheitlich von „Polypill“ gesprochen.

## Evidenzlage

Die meisten Studien zur „Polypill“ untersuchen in erster Linie deren Effektivität bezüglich der Therapieadhärenz sowie hinsichtlich der Kontrolle von Risikofaktoren wie systolischem bzw. diastolischem Blutdruck (SBD, DBD) und Blutfetten (insbesondere LDL-Cholesterin). In einer 2021 veröffentlichten Metaanalyse von 44 Studien bei Patienten mit Hypertonie wurde ein signifikanter Vorteil der Fixkombination hinsichtlich der Therapieadhärenz im Vergleich zur losen Kombination beobachtet [11]. Darüber hinaus waren sowohl der SBD als auch der DBD nach 12 Wochen unter Fixkombination signifikant verbessert. Eine Fixkombination aus Telmisartan (20 mg), Amlodipin (2,5 mg) und Chlortalidon (12,5 mg) im Vergleich zur Standardbehandlung wurde 2018 in einem randomisierten Open-label-Design an 700 Patienten mit Hypertonie (Blutdruck > 140 mm Hg/> 90 mm Hg), Diabetes oder chronischer Nierenerkrankung untersucht. Nach einem 6‑monatigen Follow-up waren 70 % der Polypill-Patienten auf den Zielwert eingestellt gegenüber 55 % bei Standardbehandlung (RR 1,23; 95 %-Konfidenzintervall [KI] 1,09–1,39; *p* < 0,001). Der Blutdruck lag nach 6 Monaten im Mittel bei 125/76 mm Hg bei der „Polypill“ vs. 134/81 mm Hg bei Standardbehandlung (adjustierter Unterschied: SBD −9,8 mm Hg; 95 %-KI −7,9–11,6 mm Hg; DBD −5,0 mm Hg; 95 %-KI −3,9–6,1 mm Hg; *p* < 0,001; [12]). Die Kombination von 4 niedrig dosierten Wirkstoffen (Irbesartan 37,5 mg, Amlodipin 1,25 mg, Indapamid 0,625 mg, Bisoprolol 2,5 mg) im Vergleich zur initialen Monotherapie in der Standarddosis (Irbesartan 150 mg) wurde in der QUARTET-Studie untersucht [13]. In dieser klinischen Studie konnten eine signifikante Verbesserung des SBD um 6,9 mm Hg (95 %-KI 4,9–8,9 mm Hg; *p* < 0,001) und eine signifikant verbesserte Blutdruckkontrolle der Teilnehmer unter „Polypill“ festgestellt werden (76 % vs. 58 %; RR 1,30; 95 %-KI 1,15–1,47; *p* < 0,001). Auch war in der Fixkombinationsgruppe seltener als in der Kontrollgruppe eine Dosissteigerung der Medikamente erforderlich (*p* < 0,001). Dieser Effekt war nach 52 Wochen noch ausgeprägter (SBD-Reduktion: 7,7 mm Hg; 95 %-KI 5,2–10,3 mm Hg; Kontrollrate: 81 % vs. 62 %; RR 1,32; 95 %-KI 1,16–1,50) bei gleicher Häufigkeit unerwünschter Arzneimittelwirkungen in den beiden Behandlungsarmen.

Die Überlegenheit der „Polypill“ hinsichtlich der Kontrolle von Risikofaktoren ist recht eindeutig

**Figure 2:**
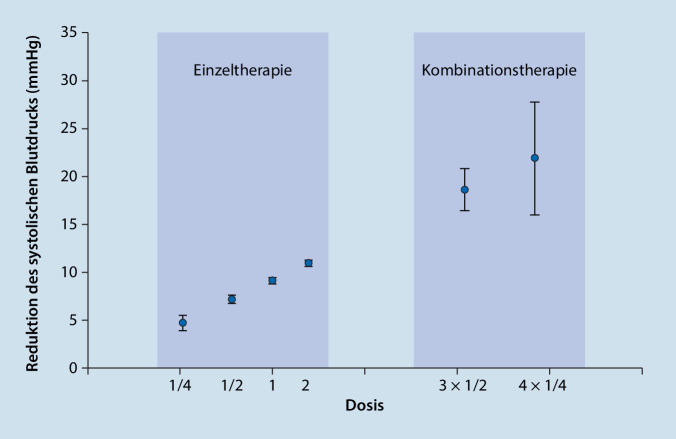

Die Evidenz zur überlegenen Wirksamkeit der „Polypill“ hinsichtlich der Kontrolle von Risikofaktoren und einer besseren Therapieadhärenz gegenüber losen Tablettenkombinationen ist mittlerweile recht eindeutig. In einer Übersichtsarbeit aus dem Jahr 2019 stellen Chow u. Meng [14] sehr ausführlich die Evidenz aus kontrollierten Studien zu dieser Wirksamkeit insbesondere in der Primärprävention kardiovaskulärer Erkrankungen dar (Abb. 2).

Dem gegenüber war die Evidenz zur Verbesserung harter klinischer Endpunkte (Sterblichkeit, nichttödliche kardiovaskuläre Ereignisse) bis vor Kurzem weniger eindeutig. In einem Cochrane-Review aus dem Jahr 2017 wurden 13 Studien mit 9059 Patienten aus 32 Ländern zusammengefasst, von denen 5 Studien auch den Zusammenhang mit der Gesamtmortalität untersuchten [15]. Die Autoren fanden keine Hinweise auf eine Wirksamkeit der „Polypill“ im Vergleich zur Kontrolle für Mortalität oder nichttödliche kardiovaskuläre Ereignisse. Gleichzeitig berichten die Autoren jedoch über eine sehr geringe Ereignisrate von etwa 1 % im mittleren Follow-up-Zeitraum (9–23 Monate), eine fehlende Power hinsichtlich der Outcome-Analysen sowie eine hohe statistische Heterogenität der Studien. Insgesamt werten die Autoren die Ergebnisse daher als nicht aussagekräftig hinsichtlich der Mortalitätseffekte einer Polypill-Therapie.

### Studien zur Primärprävention

Die erste groß angelegte Studie, die das Konzept der „Polypill“ auf harte klinische Endpunkte untersucht hat, war die 2019 erschienene PolyIran-Studie [16]. In dieser clusterrandomisierten Kohortenstudie wurden 6838 Teilnehmer zwischen 40 und 75 Jahren 1:1 in Gruppen mit Polypill-Therapie (Enalapril 5 mg, ASS 81 mg, Hydrochlorothiazid 12,5 mg und Atorvastatin 20 mg) oder „minimal care“ randomisiert und verglichen. Bei Auftreten eines Enalapril-spezifischen Hustens konnte in der Polypill-Gruppe auf eine andere „Polypill“ mit Valsartan (40 mg) umgestellt werden. Die Minimal-care-Gruppe wurde mit nichtmedikamentösen Präventionsmaßnahmen betreut, vor allem mittels Aufklärung über eine gesunde Lebensweise. In der 60-monatigen Nachverfolgung erlitten den primären Endpunkt (kardiovaskuläre Ereignisse: Hospitalisierung wegen eines akuten Koronarsyndroms, tödlicher Herzinfarkt, plötzlicher Herztod, Herzinsuffizienz, Revaskularisierung sowie tödlicher und nichttödlicher Schlaganfall) 8,8 % der Patienten in der Minimal-care-Gruppe gegenüber 5,9 % der Polypill-Gruppe (Hazard Ratio [HR] 0,61; 95 %-KI 0,49–0,75), entsprechend einer relativen Risikoreduktion (RRR) von 39 % zugunsten der „Polypill“. Der größte Einfluss konnte beim sekundären Endpunkt des tödlichen Schlaganfalls festgestellt werden, hier betrug die RRR 62 % (adjustierte HR 0,38; 95 %-KI 0,18–0,82; *p* = 0,013). Die Gesamtmortalität wies eine nichtsignifikante Reduktion von 7 % zugunsten der „Polypill“ auf (adjustierte HR 0,93; 95 %-KI 0,77–1,11; *p* = 0,43). Bei alleiniger Betrachtung jener Teilnehmer der Polypill-Gruppe, die eine hohe Adhärenz aufwiesen, fiel die Reduktion der „major adverse cardiovascular events“ (MACE) im Vergleich zur Minimal-care Gruppe mit einer RRR von 57 % noch deutlicher aus (adjustierte HR 0,43; 95 %-KI 0,33–0,55). Aus ihren Ergebnissen schlossen die Autoren, dass die Polypill-Strategie effektiv in der Prävention kardiovaskulärer Ereignisse ist und besonders in Ländern mit niedrigem bis mittlerem Einkommen eine zusätzliche effektive Strategie zur Verhinderung von schwerwiegenden kardiovaskulären Ereignissen sein kann.

In einer weiteren groß angelegten RCT wurde der Einfluss einer „Polypill“ mit oder ohne ASS bei Personen ohne vorherige kardiovaskuläre Vorerkrankung, aber mit erhöhtem kardiovaskulärem Risikoprofil (gemäß INTERHEART Risk Score) untersucht [17]. In der TIPS-3-Studie wurden in einem randomisieren Design 5713 Patienten über einen mittleren Verlauf von 4,6 Jahren beobachtet. Verglichen wurden eine „Polypill“, die Simvastatin (40 mg), Atenolol (25 mg), Hydrochlorothiazid (25 mg) und Ramipril (10 mg) enthielt, mit Placebo sowie „Polypill“ + ASS (75 mg) mit Doppelplacebo. Als primärer Endpunkt wurde ein Komposit bestehend aus kardiovaskulärem Tod, Herzinfarkt, Schlaganfall, Reanimation, Herzinsuffizienz oder Revaskularisierung gewählt, zusätzlich wurden Blutdruck und Cholesterin untersucht. Auch hier konnte für die „Polypill“ eine signifikante Verbesserung im Vergleich zu Placebo festgestellt werden. Noch deutlicher fiel die Risikoreduktion aus, wenn zusätzlich zur „Polypill“ ASS gegeben wurde.

### Studien zur Sekundärprävention

All den vorher genannten Studien ist gemein, dass sie im Wesentlichen in der Primärprävention angesiedelt sind. Auch die eigentlich in einem gemischten Setting aus Primär- und Sekundärprävention angesiedelte PolyIran-Studie kann lediglich eine Quote von 10,8 % Probanden mit vorherigem kardiovaskulärem Ereignis aufweisen [18]. In einer systematischen Übersichtsarbeit und Metaanalyse aus dem Jahr 2022 untersuchten Rao et al. [19] bis einschließlich Februar 2021 veröffentlichte RCT zum Thema „Polypill“. In der Gesamtanalyse konnte für die „Polypill“ eine signifikante Reduktion der Gesamtmortalität (RR 0,90; 95 %-KI 0,81–1,00) sowie eine nichtsignifikante Reduktion bezüglich der MACE (RR 0,85; 95 %-KI 0,70–1,02) festgestellt werden. Bei einer Unterteilung in Studien zur Primärprävention und Studien zur Sekundärprävention zeigte sich dieser Therapieeffekt für die Reduktion der MACE nur bei Studien der Primärprävention (RR 0,70; 95 %-KI 0,62–0,79), jedoch nicht bei solchen der Sekundärprävention (RR 1,10; 95 %-KI 0,82–1,47). Für die Daten der Gesamtmortalität konnte keine solche Differenzierung vorgenommen werden. Die Frage nach einem Nutzen der „Polypill“ in der Sekundärprävention konnte dementsprechend nicht beantwortet werden.

Die SECURE-Studie schließt die Evidenzlücke zum Nutzen der „Polypill“ in der Sekundärprävention

Durch die beim Kongress der European Society of Cardiology (ESC) im August 2022 vorgestellte SECURE-Studie (Secondary Prevention of Cardiovascular Disease in the Elderly) kann diese Evidenzlücke nun erfolgreich geschlossen werden [20]. SECURE ist die erste prospektive, randomisierte Phase-III-Studie, die den klinischen Nutzen einer „Polypill“ in der Sekundärprävention bei Patienten nach einem Herzinfarkt aufzeigt und hinsichtlich harter klinischer Endpunkte untersucht. Die vom Förderprogramm Horizon 2020 der Europäischen Union unterstützte Studie wurde in insgesamt 7 europäischen Ländern (Tschechische Republik, Frankreich, Deutschland, Ungarn, Italien, Spanien, Polen) durchgeführt. Patienten über 65 Jahre mit einem Herzinfarkt, der maximal 6 Monate zurücklag, mussten mindestens einen weiteren Risikofaktor aufweisen: Diabetes mellitus, Nierenfunktionsstörung, vorheriger Schlaganfall, Herzinfarkt oder koronare Revaskularisierung. Die Patienten wurden in Gruppen mit Polypill-Therapie (*n* = 1237; ASS 100 mg, Ramipril 2,5 mg, 5 mg oder 10 mg und Atorvastatin 20 mg oder 40 mg) oder Standardtherapie (*n* = 1229) randomisiert. Standardtherapie war definiert als die Behandlung, die gemäß den aktuellen ESC-Leitlinien und nach ärztlichem Ermessen in den jeweiligen Ländern die gängige Behandlungspraxis für Patienten nach Infarkt war. Das durchschnittliche Alter der Patienten lag bei 76,0 Jahren, der Frauenanteil lag bei 31 %.

Zu betonen ist, dass in der Studie keine fixierte Polypill-Dosierung verwendet wurde, sondern eine Anpassung der Dosis von Ramipril und Atorvastatin entsprechend den klinischen Werten von Blutdruck und Cholesterin während der Studie möglich war. Der überwiegende Teil der Patienten in der Polypill-Gruppe erhielt die 40 mg-Dosierung von Atorvastatin, während 40,4 % der Patienten der Standardtherapiegruppe eine potentere Statindosis erhielten. Als primärer Endpunkt wurde ein Komposit aus kardiovaskulärem Tod, nichttödlichem Herzinfarkt, nichttödlichem Schlaganfall und akuter Revaskularisierung gewählt. Der sekundäre Endpunkt war ein Komposit aus kardiovaskulärem Tod, nichttödlichem Herzinfarkt und nichttödlichem Schlaganfall. Die Therapieadhärenz wurde nach 6 und 24 Monaten mithilfe der Morisky Medication Adherence Scale (MMAS) abgefragt. Laborparameter wurden nach 6, 12 und 24 Monaten erhoben.

Nach einer mittleren Nachbeobachtungszeit von 3 Jahren konnte ein Ereignis des primären Endpunkts bei 118/1237 (9,5 %) Patienten der Polypill-Gruppe und bei 156/1229 (12,7 %) Patienten der Standardbehandlungsgruppe festgestellt werden. Dies entspricht einer RRR von 24 % (HR 0,76; 95 %-KI 0,60–0,96; *p* < 0,001 für Nichtunterlegenheit; *p* = 0,02 für Überlegenheit). Für den sekundären Endpunkt konnte eine Risikoreduktion von 30 % („Polypill“ 8,2 % vs. Kontrollgruppe 11,7 %; HR 0,70; 95 %-KI 0,54–0,90; *p* = 0,005) beobachtet werden. Bei der Betrachtung der Einzelkomponenten des kombinierten Endpunkts zeigte sich, dass alle Komponenten zum Behandlungseffekt beitrugen. Bei Auswertung der a priori definierten Subgruppen (Alter, Geschlecht, Land, Diabetes, Nierenfunktionsstörung, vorheriges vaskuläres Ereignis) zeigte sich eine beeindruckende Konsistenz des Behandlungseffekts durch die „Polypill“ über nahezu alle Subgruppen hinweg. Damit ist SECURE die erste Studie, die in einem Setting der Sekundärprävention einen klinischen Nutzen der Polypill-Therapie nachgewiesen hat.

Die Adhärenz zeigte sich ebenfalls signifikant verbessert, sowohl nach 6 Monaten (RR 1,13; 95 %-KI 1,06–1,20) als auch nach 24 Monaten (RR 1,17; 95 %-KI 1,10–1,25). Bezüglich der erhobenen Zielwerte für Blutdruck und Cholesterin konnten keine Unterschiede zwischen den beiden Gruppen festgestellt werden. Die Autoren führen als Erklärung die relativ geringen Ausgangswerte bei Studienbeginn an und verweisen auf die pleiotropen Effekte der Statine und ACE-Hemmer über den eigentlichen Effekt der LDL-Senkung bzw. Blutdrucksenkung hinaus. Zusätzlich dazu hat ASS keinen Effekt auf die Zielwerte, sehr wohl jedoch einen nachgewiesenen Effekt bei der Verhinderung kardiovaskulärer Ereignisse, insbesondere in der Sekundärprävention.

Eine zuvor publizierte retrospektive Studie, die ebenfalls eine „Polypill“ mit ASS, Ramipril und Atorvastatin in gleichen Dosierungen untersuchte, konnte neben einer Verringerung der Rate an schwerwiegenden Ereignissen auch eine signifikant verbesserte Zielwerterreichung (LDL-Cholesterin, SBD, DBD) feststellen im Vergleich mit der substanzgleichen losen Kombination mit äquipotenten Substanzen sowie mit der Standardbehandlung [21].

## Resümee und Ausblick

**Figure 3:**
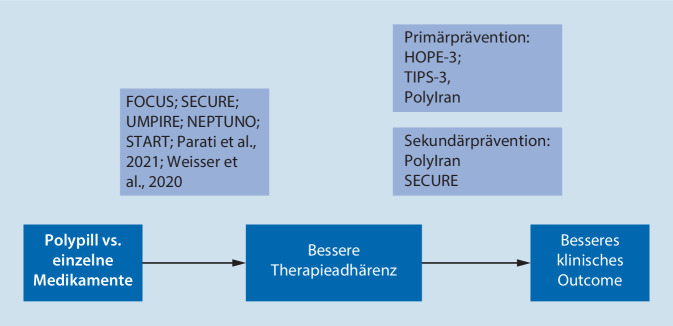

Für den Nutzen der „Polypill“ in der Primärprävention gibt es genügend qualitativ hochwertige Studien (unter anderem PolyIran, TIPS‑3, HOPE-3), die alle eine Reduktion klinischer Ereignisse bei Verwendung der „Polypill“ – im Vergleich zu Placebo oder zur Standardbehandlung – nachweisen konnten. Durch die Ergebnisse der SECURE-Studie wurde nun erstmals auch der klinische Nutzen im Sinne eines verbesserten Outcomes in der Sekundärprävention nachgewiesen (Abb. 3). Weitere Studien sind auf dem Weg, um die Ergebnisse der SECURE-Studie weiter zu untermauern. Die Ergebnisse der in Großbritannien durchgeführten PROPS-Studie, die ebenfalls in der Sekundärprävention angesiedelt ist, werden mit Spannung erwartet [22]. Der Einfluss der SECURE-Studie konnte in einer aktuellen Metaanalyse, die ausschließlich Studien in einem sekundärpräventiven Setting eingeschlossen hat, nochmals belegt werden. Hier ergab sich eine signifikante Reduktion kardiovaskulärer Ereignisse um 28 % (RR 0,72; 95 %-KI 0,63–0,82), wobei die SECURE-Studie neben PolyIran als einer der wesentlichen Faktoren für dieses Ergebnis festgestellt werden konnte [18].

Trotz der wachsenden wissenschaftlichen Evidenz und der Leitlinienempfehlungen verläuft die Umsetzung der Polypill-Therapie in Deutschland nur schleppend. Es konnte anhand von Daten des Deutschen Arzneiprüfungsinstituts (DAPI) gezeigt werden, dass trotz der erfolgten Leitlinienempfehlung zum Einsatz von Polypill-Tabletten in der Hypertonie [2] ein Rückgang in den Verordnungen zwischen 2016 und 2020 stattgefunden hat [23]. In einem aktuell erschienenen Kommentar der beiden ehemaligen Präsidenten der World Heart Federation (WHF) Salim Yusuf und Fausto Pinto wird auf die noch unzureichende Umsetzung hingewiesen, verbunden mit einem eindringlichen Appell [24]. Dabei soll die „Polypill“ nicht als neues Medikament angesehen werden, sondern als eine kostengünstige Strategie, um effektiv kardiovaskuläre Ereignisse weltweit zu verhindern. Hochrechnungen der Autoren legen nahe, dass bei nur 50 %iger Umsetzung der Polypill-Strategie eine Reduktion um bis zu 2 Mio. kardiovaskuläre Todesfälle sowie 4 Mio. kardiovaskuläre Ereignisse erreicht werden kann.

Der Polypill-Ansatz ist längst von einer Komfortmaßnahme zum wirkungsvollen Therapiekonzept geworden

Die Polypill-Therapie ist längst von einer Komfortmaßnahme für eine bequemere Medikamenteneinnahme zu einem wirkungsvollen Therapiekonzept gewachsen, wobei nüchterne Daten nicht nur eine Verbesserung der Therapieadhärenz belegen. Das Konzept führt auch zu einer überlegenen Effektivität der Behandlung kardiovaskulärer Risikofaktoren und damit zu einer Senkung der Mortalität und anderer klinischer Ereignisse [25].

Die konsequente bzw. konsequentere Anwendung von Polypill-Konzepten sowohl in der Primär- als auch in der Sekundärprävention kardiovaskulärer Erkrankungen ist nicht nur angesichts einer angenehmeren Therapiegestaltung für die Patienten sinnvoll, sondern auch aufgrund eines geringeren Risikos akuter Ereignisse, einer Senkung der Mortalität und schließlich auch einer kostenbewussten Behandlung.

## Fazit für die Praxis

- Die Polypill-Therapie hat sich längst von einer Komfortmaßnahme zu einem wirkungsvollen innovativen Therapiekonzept entwickelt. Neben einer Verbesserung der Therapieadhärenz führt das Konzept auch zu einer überlegenen Effektivität der Behandlung kardiovaskulärer Risikofaktoren und damit zu einer Senkung der Mortalität und anderer klinischer Ereignisse.
- Der klinische Nutzen der „Polypill“ in der Primärprävention ist gut belegt. Eine Reduktion klinischer Ereignisse im Vergleich zu Placebo oder einer Standardbehandlung wurde nachgewiesen.
- Mit der SECURE-Studie wurde nun auch ein verbessertes Outcome in der Sekundärprävention gezeigt.
- Dennoch verläuft die Umsetzung in Deutschland nur schleppend – ein Umstand, der angegangen werden muss.
